# Transformation of the concepts and practice of total pain and total care: 30 years of Danish hospices

**DOI:** 10.3389/fsoc.2023.1145131

**Published:** 2023-05-12

**Authors:** Helle Timm

**Affiliations:** ^1^National Institute of Public Health, Faculty of Health Sciences, University of Southern Denmark, Copenhagen, Denmark; ^2^Center for Health Care, Rigshospitalet, Copenhagen, Denmark; ^3^Faculty of Health Sciences, University of the Faroe Islands, Tórshavn, Faroe Islands

**Keywords:** suffering, relief, death and dying, society, history, institutional logics

## Abstract

The concept of total pain endeavors to encompass central aspects of suffering in relation to severe disease, death and dying. Dame Cicely Saunders introduced the concept in the early 1960s in relation to care for the terminally ill and dying patients with cancer. An examination of Danish palliative care, particularly Danish hospice care, indicates that total pain continues to be a relevant concept today. To further explore the current relevance of total pain the study examines its underlying ontology, epistemology and methodology. The study also addresses how the understanding and practice of total pain theory has developed throughout its history, in addition to how the understanding of concepts and practices is constantly being negotiated, shaped and transformed in relation to changes in society and by individuals, groups and organizations. The first of 21 hospices in Denmark opened in 1992 and they represent a case in point for exploring the transformation of total pain and total care since then. The empirical data, which are based on materials relevant to the history of the hospice movement and practice in Denmark, include national policy documents, local yearbooks, mapping, research, documentation of practice, interviews and on-going dialogue with management and staff at Danish hospices over the last 25 years. The study, which takes an abductive analytical approach, draws on my own experiences and empirical data, in addition to the empirical and theoretical research of others but also gains inspiration from a theoretical institutional logic perspective. Research shows that there are three main co-existing and interrelated institutional logics in the history of Danish hospices: care, medicine and governance. Based on inspiration from sociological and philosophical palliative care research and data on the development of Danish hospices, this study demonstrates how the concepts and practices of total pain and total care have been transformed in the compromises made due to the co-existence of these competing logics.

## 1. Introduction

Researchers have recently asked if the concept of total pain, as introduced by Dame Cicely Saunders in the 1960s, is still relevant in the 21^st^ century (Krawczyk and Richards, 2018; Krawczyk et al., 2018). According to research in Great Britain and on hospice care in Denmark, total pain and total care as concepts and practices are still relevant to the organization of and professional work being conducted today in palliative care for people suffering from life threatening diseases (Krawczyk and Richards, 2018; Graven and Timm, 2019; Graven et al., 2021). This makes sense when concepts and practices are viewed as constantly being negotiated, shaped and transformed; hence the understanding and meaning of total pain and total care are necessarily different than when they were introduced more than 60 years ago.

### 1.1. Total pain

Saunders, an English doctor, nurse and social worker, developed the concept of total pain and defined it as comprising four overall dimensions: physical, mental, social and existential pain. According to English sociologist and historian Clark (1999), her definition was developed based on years of empirical exploration and practice before being introduced as the concept total pain in 1964.

Clark (1999, 2016, 2018) writings on the subject are essential to the rudimentary understanding of the concept. He reminds us that the ontological and epistemological foundation of total pain consist of more than the four dimensions since it is crucially linked to the narrative of the person who is sick, as well as to total care, or what can be described as multiple interventions. Clark (2016) presents the following understanding of total pain:

A striking feature of Saunders's early work is its articulation of the relationship between physical and mental suffering. This reached full expression with the concept of total pain, which was taken to include physical symptoms, mental distress, social problems, and emotional difficulties []. Crucially, it was tied to a sense of narrative and biography []. This was an approach that saw pain as the key to unlocking other problems, and as something requiring multiple interventions for its resolution (Clark, 2016, p. 131).

The concept of total pain grew out of concrete empirical experience and an inductive approach, as well as research about e.g., pain and alleviation. Empirically, the concept builds on more than a thousand conversations between Saunders and seriously ill and terminally ill patients with cancer. Clark (1999) also maintains that:

This was essentially an empirical method rather than driven by theory and ideology [] over the period and as it was articulated through day to day clinical practice, it was captured increasingly within a single concept that held together a number of central concerns. By such means the method was to become a theory (Clark, 1999, p. 732).

### 1.2. Developing into palliative care and palliative medicine

Although the concept of total pain is not mentioned explicitly, it nonetheless colors international and national definitions of palliative care that involve the four dimensions of suffering, as with the most recent definition from the World Health Organization (WHO), (Sepúlveda et al., 2002), which the Danish health authorities have translated and disseminated (Danish Health Authority, 2017), and also the latest definition from the International Association for Hospice and Palliative Care (IAHPC), (Radbruch et al., 2020). In the WHO definition the response is called total care, while in the IAHPC definition it is called holistic care. None of these definitions, however, explicitly mention the narrative or biography of the person who is ill as crucial to understanding total pain or in offering total care.

While hospice philosophy and practice has inspired definitions, organization, and practice in terms of the development of the speciality and sub-speciality of palliative care, theoretical development of the concept of total pain has been different:

[] research about total pain in clinical practice and policy is limited and total pain, despite its central place in the history of palliative care, remains remarkably under-researched, conceptually and empirically. Consequently, engaging with total pain in clinical practice lacks a secure theoretical and analytical foundation (Timm et al., 2021, p. 12).

How much this lack of a firm theoretical and analytical foundation underpinning total pain is viewed as a problem, however, varies according to methodological standpoint. Researchers who primarily work using an empirical and phenomenological approach will not necessarily perceive this deficiency as problematic. In an English context Gunaratman (2012) states, that “Total pain is a multimodal method of auscultation and care as much as it is a philosophy [] which is ontologically and temporally insecure and/or withdrawn” and that this openness provides scope for a performative total care practice in concrete temporal and spatial contexts. This view is backed up by recent empirical studies at Danish hospices (Graven and Timm, 2019; Graven et al., 2021).

However, if we adopt a critical sociological approach, it is legitimate to ask questions about the concrete, historical conditions for practicing the concept of total pain, listening to patient stories and providing across-the-board total care. Or about inequality and power: between professions, institutions, doctors and scholars. Or about the economic, managerial and administrative framework for practicing palliative care.

Hospice philosophy and the core concepts of total pain and total care have developed into the field of palliative care, which is primarily a medical field, defined as a medical specialty or sub-specialty. In line with this, sociologists like Clark and Seymour (1999) have asked to what degree the field of palliative care would be able to resist a growing medicalisation as well as the routinisation and the bureaucracy of the modern health care system.

Small and Gott (2012), who point out that the relevance of concepts and practice transform over time, looked into the contemporary relevance of another concept in the same field, namely the concept of dying awareness, as introduced by Glaser and Strauss (1965). Small and Gott (2012) found that the relevance of dying awareness was replaced by a focus on dying a good death:

This revivalist approach links with a wider construction, “the good death”, in which the informed and involved patient receives the total care for total pain that is the mantra of the modern hospice movement (Clark, 2002). The good death has replaced open awareness (after incorporating it) as the aspired for normative (Small and Gott, 2012, p. 364).

Addressing the issue of whether total pain and total care continue to be relevant from a sociological perspective requires examining society, the health care system in general, organizations, interest groups and individuals over time.

The changing picture of death and dying [] underlines the strengths of Glaser and Strauss' thesis and highlights shortcomings when their insights are applied to the present day. These shortcomings are the product of changes in the epidemiology of dying, different sorts of policy and practice especially the development of palliative care, and changes in attitudes both at a general societal level and in terms of the relationship between health care providers and patients (Small and Gott, 2012, p. 365).

The concept of total pain developed in certain time and space. Day-to-day clinical practice, treatments, professionals and patients–everything—seem to differ dramatically nowadays from the health care context of care for the dying in the 1950s and 1960s. To explore the contemporary meaning and significance of the concept of total pain, this study focuses on total pain and total care in the history and practice of Danish hospices 1992–2022, as a case in point.

### 1.3. Danish hospices and Danish health care

Denmark has a population of around 5.8 million people, and since the first Danish hospice opened in 1992 the number has grown to 21, 19 for adults and two for children up to 18 years of age. All Danish hospices are currently specialized palliative care institutions, and the adult hospices have 256 inpatient beds that meet the European Association of Palliative Care recommendations (Radbruch and Payne, 2009, 2010). Only four hospices, all of which are situated in the Capital Region of Denmark, have an outpatient function. There are not any Danish hospices that offer day care facilities. To become a specialized palliative care unit in Denmark, the main purpose of the units must be palliative care and they are required to employ nurses and physicians, in addition to having at least two other kinds of professionals, all with specialized palliative care skills (Danish Health Authority, 2017). Apart from the 21 hospices, Denmark has 30 other specialized palliative care units in the form of hospital wards and teams (Jarlbæk, 2021).

Danish hospices are self-governing institutions but have been included in the publicly funded health care system since 2000. Hospices fall under the jurisdiction of the Hospital Act and have operational agreements with the regional authorities.

Danish health care is embedded in the public Danish welfare system, which has been described as a specifically Scandinavian model that is different from more liberal models. In 2007 the Danish health care system was re-organized into fewer regions (five), which are responsible for hospital treatment at a special and a basic level, with fewer municipalities (98) having responsibility for, e.g., care at a basic level. The 2007 local government reform led to the de-centralization of care as well as to an increased centralization and specialization of treatment in fewer but bigger hospitals.

All in all, over the 30-odd years which this article concerns, the Danish welfare state has developed in a more liberal direction and, some argue, it can now be characterized as a competitive state (Pedersen, 2011). Over this period the Danish health system has increasingly adopted characteristics of international neo-liberal ideals such as those pursued by new public management and new public governance; competition between entities, including more widespread privatization and outsourcing, is seen as a driving force toward efficiency and quality. This competition is accompanied by documentation of efficiency, quality and outcome, including evidence-based practice, with efficiency being linked to specialization and specialized treatment provided in increasingly shorter programmes at centralized hospitals or in the private sector, while more and more areas such as rehabilitation and care are being entrusted for the most part to the municipalities. Supply and demand are essentially based on the users' free choice of services, which in turn places greater focus on individual responsibility for health, sickness, quality of life and death.

To conclude Danish hospices are based on an international hospice philosophy and the core concepts of total pain and total care, including the institutional logics of care and medicine. Over the last 30 years society, the health care system, treatment options, the role of professionals and patients, as well as palliative care have developed dramatically. A third logic, one of policy and governance, also gained a position among the other two. Consequently, the main two questions that this article asks are: how does this relate to the understanding, practice and vocabulary concerning total pain and total care in Danish hospice institutions over time? How do institutional logics of care, treatment and governance relate to the appearance, constitution, changes and status of Danish hospice institutions?

## 2. Material and methods

### 2.1. Material

The study draws on different types of material, relevant to describe and understand Danish hospice history in relation to the concepts of total pain and total care; policy documents, texts on the genesis of hospices in Denmark, annual yearbooks and anniversary publications, national mappings, research, documentation of practice at hospices, data from two focus group interviews and an ongoing dialogue between the author and leaders and staff at Danish hospices during the last 25 years.

### 2.2. Analysis

The analysis is inspired by the perspective of institutional logic. The theory of institutional logic provides inspiration for linking the analysis of concepts and actions relating to hospices, total pain and total care across individuals, organizations and societal structures.

Thornton and Ocasio (2008) define an institutional logic as the socially constructed, historical patterns of cultural symbols and material practices, including assumptions, values, and beliefs, by which individuals and organizations provide meaning to their daily activity, organize time and space, and reproduce their lives and experiences (Thornton et al., 2013, p. 2).

Thornton et al. (2013) assert that there are:

[] four fundamental metatheoretical principles of the institutional logics perspective: the duality of agency and structure, institutions as material and symbolic, institutions as historically contingent, and institutions at multiple levels of analysis (Thornton et al., 2013, p. 6).

The four principles guided the analysis of the empirical data and structured the presentation of the findings, first and foremost the logics of care, medicine, and governance at play. Although the four theoretical principles are co-existent and overlapping, in this study the principle of historic contingency has been dominant, assuming a chronological order, moving from the past to the present over a brief period of 30 years.

The analytical framework for this study is abductive (Timmermans and Tavory, 2022) in the sense that it analyses history and empirical data inductively while simultaneously including theory and advancing further, as new understandings grow from data and make room for new questions.

## 3. Results: total pain and total care in Danish hospices, track and changes

### 3.1. Hospices as historic contingent

The meta-theoretical principle of historic contingency has to do with the relevance, interpretation and meaning of institutions and core terms historically over time:

Modern societies are typically more influenced by the logics of the state, the professions, the corporation, and the market (Thornton et al., 2013: 12).

Danish hospices in the present day seem to have shifted away from being mainly rooted in the logic of care to being characterized by a mix of three overarching logics: care, medicine and governance.

#### 3.1.1. Rooted in private institutions and the logic of care

The hospice philosophy and its core concepts of total pain and total care was an important source of inspiration for the work at the first Danish hospices and in many ways a natural extension of the Christian tradition of charity and care in previous foundations.

The first hospice in Denmark, Sct. Lukas Hospice, opened its doors in 1992, followed by one in 1995, one in 1997, and two in 1999. Thus, the first five hospices were established in the 1990s, three of them rooted in religious foundations, and four were established within the physical setting of those.

In earlier days, the Danish health system also included work carried out by nuns from Catholic and Protestant religious orders in connection with practicing in and establishing, e.g., hospitals, home nursing and education. During the 1970s and 1980s the private foundations and the nuns were more or less phased out and replaced by modern, public and secular institutions of the welfare state (Malchow, 2013). Sct. Lukas Stiftelsen, which was one of those foundations had hitherto run a hospital and nursing training, and now searched for a new mission.

In the 1990s, the first decade of Danish hospice history, the hospices worked as private organizations, recognized by, but not part of, the public health care system. Access was free regarding to referrals but had to be paid for privately or supported by the local authorities or financed by the founders.

The manager of the first hospice, like the ones to follow, was a nurse, and described the way of working like this:

a new way of interacting with and providing care for the incurably ill and their families; a basic principle that puts the patient—not their illness—at the center; underlying Christian values being pivotal; self-referral; everyone basically being welcome at the hospice (Hee, 2012, p. 16–19, my translation).

#### 3.1.2. Integrated into the public health system—And recognized as specialized units

The second decade of the history of hospices in Denmark was crucial regarding their further development and status. During this decade the hospices were formally recognized and integrated into the public health service and were accorded the status of specialized institutions. Also, they received further private funding and support from the public, and mushroomed, with a further nine hospices being set up and existing hospices expanding their capacity.

The hospices received political attention. The first national recommendations for palliative care in Denmark were issued in 1999 (Danish Health Authority, 1999). In 2000, for the very first time, hospices were granted funds via the National Budget. As part of new health legislation in 2005, each of the regions undertook to enter into an operating agreement with at least one hospice, and the following year the National Budget allocated funds for the establishment of new hospices and the first 2 years of their operation (Videbæk, 2012).

As the hospices were incorporated into the public health service AND defined as specialized palliative entities, this involved integration into a medical structural logic.

Nonetheless, the hospices continued as autonomous institutions with a certain amount of managerial and financial leeway, private governing boards, support groups, etc. and generally defined through national recommendations stemming from 1999 (Danish Health Authority, 1999).

Since 2010 a further five adult hospices and two children's hospices have been established. Apart from one advocacy group that still says that it campaigns for more hospice places (hospiceforum.dk), there are no loud calls for more hospices in Denmark.

#### 3.1.3. Governed and managed between logics

Over the last three decades hospices have become a more integral part of specialized palliative care institutions in the Danish health care system. But then again, perhaps not quite. An urgent topical discussion in Danish hospices concerns palliative care, which is now dominated more by treatment and life-prolonging technologies than previously, which can be a dilemma in terms of total pain and total care. If more resources are devoted to medical treatment, it may be at the expense of social, emotional, and existential dimensions of pain and care.

In a previous Danish study (2017–2019) analyzing some of these issues in light of the concept of medicalisation and hope theory, we found that current management and practices at Danish hospices appear to be poised between existential hope and medical hope (Graven et al., 2021); i.e., they balance between the logic of care and the logic of medicine:

The increasing medicalization of dying was accepted pragmatically by the HM (hospice managers) as a consequence of delivering specialized palliative care. Several HM emphasized this development as “reality” and something “we have to face” (Graven et al., 2021, p. 16).

As part of the aforementioned study, two focus group interviews were conducted in 2018 with the managers of 13 out of 19 hospices and also addressed a third institutional logic: governance. The hospice managers indicate that the domain of hospices is characterized by a certain resistance but also demands from their operators, the Danish regions, and that they are partly squeezed by competition from hospital-based entities, e.g., specialized palliative departments and teams. Finally, they also indicate that the rest of the health service is under so much pressure that care for the terminally ill is postponed until the last minute.

[] in a way, you could say that the external system is forcing us to align. Take the whole LKT [learning and quality teams] project [a regional quality assurance programme in which all specialized palliative care units took part] we're supposed to align this and that treatment and all of us do such and such in one specific way. Also the regions [] the occupancy rate should be this much and there should be this number of beds for this number of patients [] (Focus group interview 1).

Hospice managers, as highlighted in the aforementioned article (Graven et al., 2021), feel that the balance between practice rooted in the hospice philosophy (total pain and total care) and medical practice (treatment of diseases or physical symptoms) is necessary and, for the most part, beneficial:

We have to be flexible, we're facing a development toward a more modern palliative care—we could choose to stay where we were and say: here is a space for spirituality and we don't want them to come here with CVC. That was where palliative care started in Denmark 25 years ago. But we have to follow people who choose hospices and they do it in a different way today (Focus group interview 1).

Another example from a hospice manager also shows support for pursuing and maintaining this balance:

We now know that palliative care—care to alleviate pain—is nationally uniform, or more or less so, because we work according to clinical guidelines. So, in that way I think that patients get the same care … the situation has changed enormously, take for example national criteria for referral. I do think that things have become more aligned [] (Focus group interview 1).

Hospice managers see this development as unavoidable, as part of new demands from patients and their families and as part of modern palliative care. However, they also imply that development and practice are caught not just between care and treatment, but also between certain demands with regard to governance. On the other hand, the managers do not reflect on what strategies have in fact led to the present situation.

### 3.2. The duality of agency and structure in the development of Danish hospices

Another meta-theoretical principle is that of the relationship between agent and structure, i.e., how are actors, action and order related and how does this lead to change:

[] the goal is to examine how action depends on how individuals and organizations are situated within and influenced by the spheres of different institutional orders, each of which presents a unique view of rationality (Thornton et al., 2013, p. 10).

At the beginning of Danish hospice history, the actors were predominantly religious institutions and individuals, mostly with a healthcare background. However, as hospices and palliative care took root and developed in Denmark, multiple players became involved—other individuals, private and professional advocacy groups, public authorities and institutions. And as individuals, collective organizations and institutions, they were differently anchored in the three overarching institutional logics.

#### 3.2.1. Structural change and visions put into action by groups of interest

Throughout the 1980s individuals and institutions were already critical about care for terminally ill cancer patients—and the institutional framework for this care (Sjøgren, 2000; Dalgaard, 2001; Raunkjær, 2008; Hee, 2012).

In an alliance between Sct Lukas Stiftelsen's board of directors, senior nuns, and an appointed experienced, senior cancer nurse, an earlier idea about establishing a hospice for seriously and terminally ill patients was realized. However, the starting point was structural changes in the form of radical modernization of the state welfare institutions such as the health service, social services and education. Even though the religious foundations and religious orders are defined here primarily through the logic of institutional care, these institutions also had considerable managerial experience across the health system (Hee and Henriksen, 2012; Christiansen and Timm, 2019).

The vision of establishing a hospice was also inspired by visits to hospices in England and the US, where the hospice movement and philosophy had been developing for a longer period. Religious orders and foundations were not the only ones to focus on better conditions for terminally ill cancer patients and their families. Several professional groups, in particular nurses, psychologists, members of the clergy and doctors with experience in cancer treatment and palliative care were active in this regard.

As early as the 1990s, advocacy groups had been set up that continued to influence developments. In 1990 the overarching body [the Association for care at the end of life] was founded and in 1992 the Nordic associations were gathered in an umbrella association bearing the same name. Throughout the 1990s these associations became the pivotal point for, e.g., cross-disciplinary conferences and courses and the exchange of experience, partly via the cross-disciplinary Nordic journal for palliative medicine, [Care: Nordic Journal of palliative medicine], which is still issued four times a year. In 2001, the association Hospice Forum Danmark^1^ was also set up with the explicit aim of coordinating the establishment of hospices and developing the hospice philosophy (Dalgaard, 2001). Finally, in 1999 an association of senior staff at the existing specialized palliative units was set up (Kopp, 2017).

During the 1980s and 1990s, central authorities and organizations produced various formal and less formal reports about palliative care for the terminally ill, which also looked at the need for organizational and educational programmes (Raunkiær 2008). While the central health authorities had tried in the 1980s and in 1996 to address the issue of hospice care and supportive care, the first national expert recommendations for pain alleviation were, as already mentioned, issued in 1999.

A key player in cancer treatment, cancer research and support for cancer patients in Denmark is the private organization Danish Cancer Association, which is the largest body active nationwide in combating disease. Throughout the 1990s and the 2000s cancer treatment came under the spotlight because of the relatively high mortality rate among Danish patients. From 2000–2016 four cancer plans were established, two of which also concerned the issue of palliative care (National Board of health, 2000, 2005, 2010; National Board of Health, 2016). Thus, there was now a political focus on the conditions for people with advanced cancer, including the relatively high mortality rate.

At the same time the chair of the advocacy group Hospice Forum Denmark during the decade of 2000 was a former member of the Danish parliament for a Christian party who had good contacts to members of parliament and the hospice environment. During her directorship, a number of questions and proposals were forwarded to the parliament from the late 1990s onwards and into the 2000s that ultimately helped to secure funds and recognition for hospices as a component of public specialized palliative care.

This could be viewed in particular as an alliance between central policy makers and advocacy groups centered on issues such as cancer treatment, palliative care and hospices. The regional operators and authorities and the newly established expert organizations, however, were initially reserved about explicitly voicing their views on hospice institutions.

At the same time, though, there was a good deal of activity in the growing field of palliative care that had implications for the hospices. Firstly, professional mono-disciplinary organizations were established, and mono-disciplinary training programmes designed. At the end of the decade, in 2009, a national knowledge center for palliative care was also set up (rehpa.dk)^2^, as well as a national quality assurance entity focusing on palliative care (dmcgpal.dk)^3^. In addition to expanding and adding new programmes, it was now time for analysis, standardization and quality assurance, efforts in which the hospices played an active part.

While the common focus in the general field of palliative care remained on total pain and total care, there was a linguistic and conceptual shift from care and pain alleviation for the terminally ill and their loved ones to palliative care and palliative medicine for people with incurable illness.

#### 3.2.2. Within the alliance of medicine and health authorities

Hospices have been carried along in this development, and with respect to the logic of medicine and the logic of governance have acted according to expectations. From the perspective of institutional logic, this development can be viewed as a strong alliance between the logic of medicine, primarily the logic of medical doctors, and the logic of governance, primarily the logic of authorities.

The fact that the logic of medicine and that of modern governance go hand in hand and draw on traditional alliances between medical science, the medical profession and the health authorities/state is nothing new. In Denmark, as presumably in most countries, it is the medical societies which have pressed ahead with efforts to develop palliative care into a specialized field, develop clinical guidelines, and carry out evidence-based medical or health service research. Other professional groups, including nurses, have followed suit and supported and managed some of these efforts, although in this endeavor, it is the doctors in particular who have been in an alliance with health authorities at national and regional levels. This alliance, which also fights its own particular battles, can be seen explicitly in the battle for palliative care as a medical specialization, in efforts to develop national and regional quality, in research subjects and methodology, in national recommendations, and in the efforts to set up mono-disciplinary medical training in palliative care. However, there is also an implicit side to overall development in the form of inclusion/exclusion of organizations, groups and individuals. Although most examples of this are not relevant to this article, I will mention a few here which specifically concern hospice management.

As already described, the senior managers of the three types of specialized palliative care entities came together to form a common management association in the 2000s. However, this association was never successful, perhaps because the managers of the hospices and the hospital-based managers and their institutions were mostly rooted in different logics. The management association was eventually disbanded, and the senior managers of the individual entities have since been represented in regional palliative care councils.

Within the last decade, hospice managers have formed their own association, which they say has led to greater dialogue and common ground between the nation's hospices. The hospice managers themselves point to their much stronger focus on management in recent years and say that they view themselves as professional managers rather than professional experts. This was not the case earlier, when hospice managers apparently viewed themselves as knowledgeable professional experts who should take the lead in developing specialized total hospice care, and who, moreover, were in charge of referrals and directly involved in the care (Focus group interview 1 and 2).

Organisationally, however, they do not fit into one particular category: the hospices neither fully belong to the public health service nor to private organizations. Neither are they fully recognized by operators, regions or doctors:

[] our closest partners, who are doctors, and who are based at the hospitals and want to try something completely different from what we have, would rather have more wards and would like to have their own palliative care section at the hospitals … it's not that they don't like going there [to the hospices]; it has absolutely nothing to do with that, but if you asked them, they'd rather not have to because they see themselves more in a hospital setting (Focus group interview 1:23).

The (female) senior nurses in management, as kind souls, so to speak, think in terms of a compromise, although some of them dream of making the hospices into professional enterprises with professional governing boards and bypassing the operators to go directly to the highest political level, namely the Minister of Health. In other words, they dream of a type of freedom like that in the first part of the history of hospices in Denmark, only with the continued status as specialized entities. It is interesting, however, that hospice nursing staff are apparently not supposed to see themselves simply as employees:

[] You can't work at a hospice without painful pangs and scars now and again, otherwise we're talking about employees, and we don't need them at hospices (Focus group interview 1:5).

Practice and management at today's hospices are thus characterized by all three institutional logics and the managers are split between them when it comes to future strategy.

### 3.3. Danish hospices as material and symbolic—Total pain and total care in practice

A third meta-theoretical principle is that institutional orders consist of both material and symbolic elements:

A key principle in the institutional logic perspective is that each of the institutional orders in society has both material and symbolic elements. By material aspects of institutions, we refer to structures and practices; by symbolic aspects, we refer to ideation and meaning, recognizing that the symbolic and the material are intertwined and constitutive of one another (Thornton et al., 2013, p. 10).

Both practically and symbolically, hospices have the status of palliative care entities providing total care in relation to death and dying. In other words, hospices aim to bridge the gap between medical treatment in a very effective and specialized health care system and all the rest—the existential, the spiritual, the social, and the aesthetic dimensions of pain and care.

#### 3.3.1. Houses for total pain, total care, death, and dying

Historically hospices build on a long tradition of care for the weakest in society, with language and actions that relate to illness and death. The first Danish hospices were placed in former convents or hospital establishments, some of which had their own church. Even if the first hospices are now being replaced by more contemporary buildings, and even if the Christian principles that were hallmarks of the first hospices have been replaced by more secular and existential principles, hospices nonetheless build on a very long tradition of care that stands for something fundamentally human and charitable: faith, hope and charity, which, as we have seen, are still prominent characteristics of modern hospice care.

This is in line with the international hospice history, originally referring to hostels and later to private “homes for the dying” established from the late 1800s up until the beginning of 1900s (Clark, 2016, p. 33). In Denmark, as in the UK, it seemed necessary for the hospice movement to establish houses outside of hospitals to make room for total care—or the logic of care, which was seen as suppressed by the logic of medicine in hospitals.

To illustrate the symbolic meaning of hospice as a house and even a home, I have to quote Clark one more time. It is kind of a myth in modern hospice history that a particular patient, David Tasma, whom Cicely Saunders took care of early in her career, was a special inspiration for her to establish the first hospice. He also left her some money for this place, apparently followed by the words: “I'll be a window in your home”. Clark writes:

It provided Cicely with an image to take her forward, an offer to accept and a vision to fulfill. The window brings light into the building. At night, the light shines out from it. The window can also be an opportunity. It provides air and ventilation. It is the meeting place of the inner and the outer, the private and the public [] (Clark, 2018, p. 59).

In the same way, Danish hospices as concrete houses and institutions have come to represent the possibility of handling total pain and offering total care when people are dying. They are houses with a focus on both an interdisciplinary effort and the care of educated and experienced nurses.

Also, Danish hospices may be seen as linking a long tradition of caring with modern and evidence based interdisciplinary medicine.

#### 3.3.2. Beautiful buildings in beautiful sceneries

At the instigation of a private foundation, Realdania, and with the participation of most of the aforementioned advocacy groups in the 2000s, a construction and design programme for building the good hospice was set up (Kleis, 2012). It was a project based on experience, and two private foundations in particular subsequently invested in architectural competitions and buildings that respected these principles. Later, experience and evidence-based knowledge were collated to make adjustments to the programme (Kjeldsen and Timm, 2012, arkitektur-lindring.dk).

Notably, local help with building plots or buildings in the appropriate location and the aforementioned organizations has helped to ensure that most Danish hospices are situated in beautiful natural, green surroundings, and are planned, built and designed according to architectural and practical principles concerning private life and relations, functionality, nature, light/sound/air/temperature and ambience (Kjeldsen and Timm, 2012, arkitektur-lindring.dk).

Thus, irrespective of whether people are aware via the media, academic studies or personal experience of the practice of total care for the terminally ill and their families at Danish hospices, the attractive establishments in pleasant surroundings dotted around the country symbolize that help is at hand in a well-managed, orderly and unpressurised environment—in spite of terminal illness, sorrow and death.

#### 3.3.3. From public skepticism to full acceptance

As mentioned several times, the modern hospice movement had its origin in dissatisfaction with the circumstances of death and dying in modern hospitals. In parallel, the death awareness movement worked for more openness about death and dying (Jacobsen and Simuyemba, 2011). Looking further into this, researchers found that the fight for death awareness was replaced by palliative care and its ideal of “a good death” (Small and Gott, 2012). My point here is that Danish hospices, as houses and institutions, played (and play) an important role in the public awareness of and openness toward death and dying as a part of life (Graven and Timm, 2019). In this way, the hospices take part in a much broader cultural change toward more open approaches to the body, sexuality, and death (Jacobsen and Simuyemba, 2011), including life-threatening diseases, dying, and bereavement addressed not only in research but also in literature, movies, theater, music etc.

During the last 30 years, the possibilities to offer total care in public health care, in general, seem to have diminished, e.g., the terms “missed care” and “rationing of care” have been introduced (Mandal et al., 2020). While the first Danish hospices were sometimes referred to as “death hospitals” (Nielsen, 2020), this is no longer the case. Studies show that, apart from dying in one's own home, Danes would prefer to die in a hospice (compared to a hospital or a nursing home) (Timm and Hagedorn-Moeller, 2013).

Unlike the situation with hospitals, nursing homes and domiciliary care, Danish hospices are apparently rarely or never exposed to criticism. In a huge undertaking, all media texts were analyzed that concern Danish hospices from 2003–2004 and from 2012–2013. All the articles described hospices positively (Markfoged, 2014). The same goes for the research referred to earlier. Everything is apparently relative, and perhaps we need, symbolically and in practice, places in the health service which offer the superlative, where peace and quiet, order and rituals prevail.

Thus, irrespective of whether people are aware via the media, academic studies or personal experience of the practice of total care for the terminally ill and their families at Danish hospices, the attractive establishments in pleasant surroundings dotted around the country symbolize that help is at hand in a well-managed, orderly and unpressurised environment. In spite of terminal illness, sorrow, and death.

### 3.4. Dilemmas and perspectives at multiple levels

The last meta-theoretical principle:

[] assumes that institutions operate at multiple levels of analysis and that actors are nested in higher order levels—individual, organizational, field and society. This assumption conforms to the empirical observation that institutions are in conflict, but also simultaneously provides constraint against and opportunities for change by actors (Thornton et al., 2013, p. 13–14).

The above analysis already appears to confirm this principle; concepts and practices like e.g., total pain and total care is constantly being negotiated, shaped and transformed in relation to society and by individuals, groups and organizations.

#### 3.4.1. An individual and single organizational everyday life level

At the level of everyday life, where hospices are explored using a phenomenological or ethnographic approach, life and death in Danish hospices seem to function well in most areas, as previous research confirms (Steenfeldt, 2013; Graven, 2015; Moestrup, 2015; Graven and Timm, 2019). Total pain and total care have been explored particularly in terms of spiritual-existential care, which seems to be an overarching aspect. Patients and their families receive holistic palliative care with a human face, something which they have often found lacking in their previous care. The staff have often chosen to work at hospices because they are associated with the unique opportunity to provide relief based on a holistic care approach.

According to national recommendations (2017), hospices, which are seen as part of a specialized approach, have a commitment to engage in research. However, it is a question of small-scale institutions with between 12 and 30 beds that do not have staff with research competencies. Thus, research at Danish hospices is conducted by external researchers, in other words people who have an interest in hospices as unique institutions and/or people who are invited to conduct their research at a hospice. Their research is not, as such, about the concepts of total pain and total care. Which might actually be inspired by Nordic philosophical and existential theory of care and caring (Martinsen, 1989, 1998; Eriksson, 1992, 2006).

The concept and practice of total pain is also challenged, for example, by a lack of doctors with specialized palliative skills. Total care is challenged by the increase in medical treatments at hospices and by new kinds of patients whose illness is more advanced and who are there for shorter periods of time, but also by a health system characterized by internal competition at specialist level and a lack of resources—a system generally under strain.

#### 3.4.2. At field level: investigating conditions and practice at institutional level

At field level, where the hospices are examined as a type of institution in the overall field of palliative care and in the health system, the challenges that are also experienced at the individual hospices are analyzed in a larger context.

The hospices went from being self-managed, small-scale, private institutions responsible for operation and finance to becoming part of the public health system. The most radical development, though, was that hospices, which based themselves on a tradition of providing shelter to the vulnerable, became defined as specialized institutions. Palliative care and thereby total pain and total care became medical specializations/sub-specializations, which gave rise to a variety of challenges. The problem is not that hospices are unable to live up to their status since they seem to be doing just that, in addition to having increasingly qualified staff. The problem is that hospices have become aligned with the treatment-based, efficient and evidence-based hospital system and have moved away from the fundamental, everyday life level in the municipalities.

At this level the hospices face various dilemmas relating to the rest of the specialized field, to the operators/regions, and to their future legitimacy. The knowledge and know-how available at hospices are not being sufficiently spread to more patient groups and to the rest of the health system.

#### 3.4.3. Societal level: centralization and specialization, mono-disciplinary vs. cross-disciplinary

By looking at overall societal development and societal changes over time, we can explain the framework in relation to which the hospices are being developed. Both treatment and education programmes have been significantly centralized and expanded during the 30-year period analyzed here. Both have changed the conditions for the cross-disciplinary practice of the concepts of total pain and total care. At the same time the health, social and education sectors have increasingly been subjected to planning and management according to neo-liberal principles of economies of scale, management and documentation as a basis for improving quality and efficiency.

Finally, certain fundamental power structures in the health system have remained the same. The neo-liberal logic of governance has been hugely expanded, but in close alliance with a medical and doctor-oriented logic centered on assuring quality in very specific ways: via medical evidence, documentation/measuring selected quality parameters, clinical guidelines and skills descriptions. With regard to hospice practice in Denmark, this has been a necessary adaptation and is seen, for example by nurse managers at hospices, mainly as a benefit, even though it has also had implications for the practice of total care.

## 4. Summary and conclusion

### 4.1. Summary

The development of Danish hospices as a type of institution underlines the overarching themes in the perspective of institutional logic. The hospices and the concepts of total pain and total care have been developed and transformed in relation to society as a whole, involving multiple actors, existing structures and as material and symbolic practice. There are various dilemmas in this development, as well as perspectives in relation to the further development of both hospice institutions and key concepts such as total pain and total care.

The hospice is still a very special kind of institution in the Danish health system. It is unusual for specialized health institutions to be run by nursing staff rather than doctors. It is also unconventional for institutions to have private governing bodies comprising both medical doctors and other professionals, support associations and volunteers from the local community, while at the same time being publicly funded and having the status of specialized units. The hospice, as a type of private institution, has been given a special place within the logic of governance of the public health system and it has used this position to practice total care for very ill patients and their relatives at the end of the patients' lives.

The study points to three specific dilemmas. It has been shown that being defined as specialized institutions has both pros and cons and that Danish hospices are caught between three logics, meaning that they could be described as quasi-integrated in the specialized public health system.

The first dilemma concerns the question of what type of institution the hospice should be in the future to practice and disseminate the concepts of total pain and total care in the Danish health system: private, public or something in between. The hospices and their managers do not have any special legitimacy in a public health system that is largely run in a traditional alliance between the medical profession, medical specializations and health authorities. The fact that hospices as institutions are managed by nursing staff and represent care more than medicine may give them a raison d'être as far as the public at large are concerned, but not as far as the health system is concerned. It will presumably be necessary to review this special status, perhaps in connection with the coming health reform of the Danish health system in 2023–2024.

The second dilemma concerns the division into a specialized and a general level. Thinking in these two levels is not part of the logic of care but, on the contrary, is part of the logic of medicine and the logic of governance. One outlook could be that the hospices retain their status as specialized institutions but develop much closer cooperation with the general municipal level. This might require a greater focus on outbound, supporting functions and cooperation when it comes to cases of ill people in their own homes or at residential nursing homes, and it might possibly also involve day and night stays at present hospices.

The third dilemma concerns how the primarily experience-based development of “the total” is developed on a more theoretical, research-oriented basis. Total pain and total care are practiced at the hospices by nurses in particular. It might be beneficial for future research examining hospices to be centered on the cross-disciplinary concept of total pain and nursing concept of total care as something that is different from and goes beyond existential nursing. Danish hospices have years of experience of approaching their work from the narrative of the sick, but it has not been explored. Finally, there is the issue of the role of nurses in practicing professional total care: does it require something other than and more than professional skills? And, how is total care different from the best of care in general?

### 4.2. Conclusion

The emergence and development of the Danish hospice as an institution seem to be dominated by three overarching institutional logics: care, medicine and governance. These three dominating logics overlap but at the same time they are contradictory and contrasting.

The logic of care, and hence total pain and total care, within hospices is mainly defined as a holistic (and patient- and family-oriented) approach that is under pressure from the other logics and that must be defended at various levels by various actors. Moreover, total care in hospices is more or less defined by highlighting existential care, which in turn also reduces research in Danish hospices to this dimension.

## Data availability statement

The data analyzed in this study is subject to the following licenses/restrictions: The data consist of two focus group interviews (2018) conducted in a former study (Graven et al., 2021), referred to in the article. Requests to access these datasets should be directed to HT, timm@sdu.dk.

## Ethics statement

Ethical approval was not required for the research drawing on previous human studies in accordance with the local legislation and institutional requirements. Written informed consent to participate in this study was not required from the participants in accordance with the national legislation and the institutional requirements.

## Author contributions

HT is the only responsible for this study. The focus group interviews referred to and cited, were conducted in relation to an earlier study and in collaboration with Dr. Vibeke Graven.

## Footnotes

^1^Hospice Forum Danmark. Available online at: www.hospiceforum.dk

^2^REHPA, the Danish Knowledge Centre for Rehabilitation and Palliative Care. Available online at: www.rehpa.dk

^3^Danish Multidisciplinaire Cancer Group for Palliative Care. Available online at: www.dmcgpal.dk
